# Thrombotic Microangiopathy and Acute Kidney Injury Induced After Intravitreal Injection of Vascular Endothelial Growth Factor Inhibitors VEGF Blockade-Related TMA After Intravitreal Use

**DOI:** 10.3389/fmed.2020.579603

**Published:** 2020-10-07

**Authors:** Ramy M. Hanna, Ngoc-Tram Tran, Sapna S. Patel, Jean Hou, Kenar D. Jhaveri, Rushang Parikh, Umut Selamet, Lena Ghobry, Olivia Wassef, Marina Barsoum, Vanesa Bijol, Kamyar Kalantar-Zadeh, Alex Pai, Alpesh Amin, Baruch Kupperman, Ira B. Kurtz

**Affiliations:** ^1^Division of Nephrology, Department of Medicine, University of California (UC) Irvine School of Medicine, Orange, CA, United States; ^2^Division of Nephrology, Department of Medicine, Long Beach Memorial Medical Center, Long Beach, CA, United States; ^3^Department of Pathology and Laboratory Medicine, Cedars Sinai Medical Center, Los Angeles, CA, United States; ^4^Division of Kidney Diseases and Hypertension, Donald and Barbara Zucker School of Medicine at Hofstra/Northwell, Great Neck, NY, United States; ^5^Division of Renal Medicine, Department of Internal Medicine, Brigham and Women's Hospital, Boston, MA, United States; ^6^School of Public Health, University of Pittsburgh, Pittsburgh, PA, United States; ^7^Division of Nephrology, Department of Medicine, University of California, Los Angeles, Los Angeles, CA, United States; ^8^Keck School of Science and Technology, School of Pharmacy, Chapman University, Orange, CA, United States; ^9^Department of Pathology, Donald and Barbara Zucker School of Medicine at Hofstra/Northwell, Great Neck, NY, United States; ^10^Department of Medicine, University of California (UC) Irvine, Orange, CA, United States; ^11^Herbert Gavin Eye Institute, Department of Ophthalmology, University of California (UC) Irvine, Irvine, CA, United States; ^12^Brain Research Institute, University of California Los Angeles (UCLA), Los Angeles, CA, United States

**Keywords:** intravitreal injections, thrombotic microangiopathy, diabetic retinopathy, vascular endothelial growth factor (VEGF), bevacizumab (avastin), ranibizumab (Lucentis), aflibercept (Eylea)

## Abstract

Vascular endothelial growth factor (VEGF) inhibition can cause worsening hypertension, proteinuria, chronic kidney injury, and glomerular disease. Thrombotic microangiopathy (TMA) and other nephrotic disorders have been reported with systemic VEGF blockade. These same agents are given intravitreally for age-related macular degeneration (AMD) and diabetic retinopathy (DR), albeit at lower doses than those given for systemic indications. Systemic absorption of anti-VEGF agents when given intravitreally has been shown consistently along with evidence of significant intravascular VEGF suppression. While worsening hypertension has only been seen in some large-scale studies, case reports show worsening proteinuria and diverse glomerular diseases. These include TMA-associated lesions like focal and segmental glomerulosclerosis with collapsing features (cFSGS). In this paper, we report three cases of TMA likely associated with the use of intravitreal anti-VEGF therapy. These patients developed the signature lesion of VEGF blockade in a 6 to 11 month time frame after starting intravitreal VEGF inhibitors. The literature is reviewed showing similar cases. Intravitreal VEGF blockade may cause these adverse events in a hitherto unidentified subgroup of patients. Well-controlled prospective observational trials are needed to determine the event rate and identify which subgroups of patients are at increased risk. A registry for patients who develop worsening hypertension, proteinuria exacerbation, and glomerular diseases from intravitreal VEGF blockade is proposed.

## Introduction

Vascular endothelial growth factor (VEGF) is intimately involved in the physiological function of the glomerulus. Endothelial cells rely on VEGF signaling as trophic signals and for control of diacylglycerol kinase epsilon (DAG-ε). DAG-ε can induce thrombosis if not tightly regulated (1–4). Podocytes rely on VEGF for cytoskeletal organization via nephrin, and trophic signaling is also mediated in podocyte cells via VEGF signaling (autocrine or otherwise) (1). This signaling system interacts with Rel-A (REL-associated protein) and prevents upregulation of renin–angiotensin–aldosterone signaling (RAAS) via the pro-inflammatory nuclear factor kappa B (NF-κB). Tyrosine kinase pathways interact with C-Maf-inducing protein (C-MIP) (1, 2, 5, 6). The blockade of this critical system has various pharmacological applications, namely, the inhibition of angiogenesis. As such, VEGF inhibition has served as a cornerstone of adjunct chemotherapeutic effects for blockage of angiogenesis, limiting tumor growth (1–3, 5, 7).

As a result of the clinical success of these agents, anti-VEGF treatments were adapted for intravitreal usage for patients with neovascularization. Age-related macular degeneration (AMD), diabetic macular edema (DME), and central retinal vein obstruction became amenable to pharmacotherapy (8, 9). Systemic blockade of VEGF leads to several well-known side effects (10–12). These include worsening hypertension, *de novo* proteinuria, renal limited thrombotic microangiopathy (TMA), and various other causes of nephrotic syndrome (13, 14).

The US Food and Drug Administration (FDA) never approved bevacizumab for intravitreal use but did approve aflibercept (Eylea®) and ranibizumab (Lucentis®) for intravitreal use. The label inserts state that the serum drug levels with intravitreal injections were 200-fold lower than the levels achieved by systemic administration, and thus, VEGF inhibition would be minimal (15, 16). However, data published by Avery et al. showed that intravitreal absorption could be significant (at or above 50% inhibitory concentration) and result in significant inhibition of systemic VEGF for days to weeks after intravitreal injections (8, 9, 17, 18).

Avery et al., Jampol et al., Rogers et al., and Zehetner et al. showed that intravitreal injections of VEGF inhibitors caused significant depletion of circulating systemic VEGF levels (8, 9, 17–21). The search for the clinical consequences of this observed VEGF depletion has been ongoing since these results were published. Various studies showed worsening blood pressure and hematological changes (22, 23). Recently, various groups found differences in mortality and post cardiovascular and cerebrovascular event mortality and morbidity (22–26) [though the results are not all in agreement (27–29)].

Glassman et al. and Kameda et al. did not find obvious population-wide effects of acute kidney injury (AKI) after intravitreal VEGF injections. There was also no evidence that all patients had worsening of proteinuria category between Kidney Disease Improving Global Outcomes (KDIGO) A1 to A3 (30, 31). A1 patients tended to stay in the A1 category and A3 patients tended to stay in A3. Bagheri et al. showed a positive change in hypertension, systemic VEGF levels, hemoglobin, and platelets, and though not statistically significant, 45% of patients showed worsening proteinuria after intravitreal bevacizumab (22). It is increasingly clear that a subgroup of patients may be experiencing these changes, and many factors are involved in modulating the response in a given patient.

Many confounding factors exist like vitreal absorption, total dose of drug, and genetics of response to VEGF blockade (1, 2). We present three cases of clear TMA with rapid decline of renal function in diabetic, hypertensive patients. These changes are clinically observed to occur after introduction of intravitreal VEGF inhibitors for the indication of diabetic retinopathy (DR). These cases demonstrate clearly that glomerular pathologies can be superimposed on a background of kidney disease due to diabetic nephropathy. See Table 1.

**Table 1:** Renal Toxicity observed with Intravitreal VEGF blockade.

**References**	**N**	**Age**	**Gender**	**Agent**	**Pathology on biopsy**
Hanna et al. (2)	4	53-82	F,F,F,M	Bev and Ran	Biopsy proven MCD, 45% increased proteinuria (NS), worsening HTN, Increased platelets
Nobakht et al. (4)	1	96	F	LucBevAflib	Biopsy proven CFSGS
Bagheri et al. (22) [study]	18/40	60.3 ±9.2y	33F, 7M	Bev	45% of patients with increased proteinuria
Rasier et al. (23) [study]	82	67.2 ± 5.2	44F, 38M	Bev	Significant increase in SBP and DBP
Chenugpasitporn et al. (32)	2	56,67	M, M	Bev	Biopsy proven MCD. Biopsy proven TMA
Diabetic Retinopathy Clinical Research Network et al. (33)	3	NR	NR	Bev	Decreased eGFR
Georgalas et al. (34)	2	51/68	F,M	Ran & Bev	Decreased eGFR
Jamrozy-Witkowska et al. (35)	1	NR	NR	NR	Decreased eGFR
Kenworthy et al. (36)	1	88	F	Bev	Increased Proteinuria
Khneizer (37)	1	74	M	Bev	Biopsy proven MGN
Morales et al. (38)	1	56	M	Ran	Increased Proteinuria, biopsy proven DN
Pelle et al. (39)	1	77	F	Ran	Biopsy proven TMA
Perez-Valdivia et al. (40)	1	54	M	Bev	Biopsy proven MCD relapse
Sato et al. (41)	1	16	F	Bev	Biopsy proven MCD relapse
Tran (42)	1	51	M	Bev	Biopsy proven AIN
Touzani et al. (43)	1	72	M	Bev	Biopsy proven TMA
Yen and Zhang (44)	1	56	M	Bev	Biopsy proven Endotheliosis/TMA changes
Hanna et al. (45)	1	38	F	BevRan	Worsening HTN and proteinuria, lessened with Ran use vs. Bev
Shye et al. (46)	3	58	M	Bev	Decreased eGFR, Biopsy proven CFSGS and AIN, and biopsy proven AIN
Chung et al. (47) [study]	53	59.8 average age	31F, 29M	Bev	Significant worsening in proteinuria after bevacizumab in already proteinuric patients
(Phadke-Hanna) (UR)	1	74	M	RanAflib	Biopsy proven CFSGS with TMA
(Hanna) CC	3	56,43,77	F, F, M	Bev x 2, Aflib x 1	Chronic TMA x 2, FSGS, Endotheliosis/Chronic TMA

*Aflib, aflibercept; AIN, allergic interstitial nephritis; Bev, bevacizumab; CC, current case; CFSGS, collapsing focal and segmental sclerosis; DBP, diastolic blood pressure; eGFR, estimated glomerular filtration rate; F, female; FSGS, focal and segmental sclerosis; HTN, hypertension; M, male; MCD, minimal change disease; MGN, membranous glomerulonephritis; N, number; NS, not significant; Ran, ranibizumab; SB, systolic blood pressure; TMA, thrombotic microangiopathy, UR, under review*.

## Methods

Documented (written) informed consent was obtained from the individuals in cases 1–3 for the publication of any potentially identifiable images or data included in this article; we endeavored to have no identifying information to be used in this report.

### Case 1

A 56 year-old Caucasian male with a history of type 2 diabetes mellitus with an elevated hemoglobin A1c (8.1%) (reference range: <5.7%) is reported. He has a history of moderate hypertension and chronic kidney disease with a serum creatinine of 0.9 mg/dl (reference range: 0.7–1 mg/dl) in 4/2018 [estimated glomerular filtration rate (eGFR) = 96 ml/min] (reference range: 90–120 ml/min). He was referred to nephrology care for proteinuria. The patient was diagnosed with DR and diabetic nephropathy with a urine microalbumin-to-creatinine ratio of 360 mg of albumin per gram of creatinine noted in early 2019 (reference range: <30 μg/mg or mg/g). When he first presented to care in late 2018/early 2019, he had not taken any non-steroidal anti-inflammatory agents and was only on proton pump inhibitor (pantoprazole), which was then switched to a histamine receptor 2 antagonist (ranitidine) after a short duration of use.

He complained of progressively blurry vision and was seen by an ophthalmologist, after which he was started on intravitreal VEGF inhibitor therapy in late 2018 to 1/2019. Intravitreal injections of bevacizumab (1.25 mg) were given in each eye (2.5 mg injected total) every 2 months until 7/2019 when he had a more severe episode of recurrent macular edema. This necessitated switching the anti-VEGF regimen to a monthly interval. This was also deemed necessary due to the development of possible early central retinal vein occlusion. According to this dosing schedule, the patient received a total of 20 mg bevacizumab between both eyes throughout 2019 [1.25 mg OU 1/2019 (2.5 mg), 1.25 mg OU 3/2019 (2.5 mg), 1.25 mg OU 5/2019 (2.5 mg), 1.25 mg OU 7/2019 (2.5 mg), 1.25 mg OU 8/2019 (2.5 mg), 1.25 mg OU 9/2019 (2.5 mg), 1.25 mg OU 10/2019 (2.5 mg), 1.25 mg OU 11/2019 (2.5 mg)].

**Figure 1:**
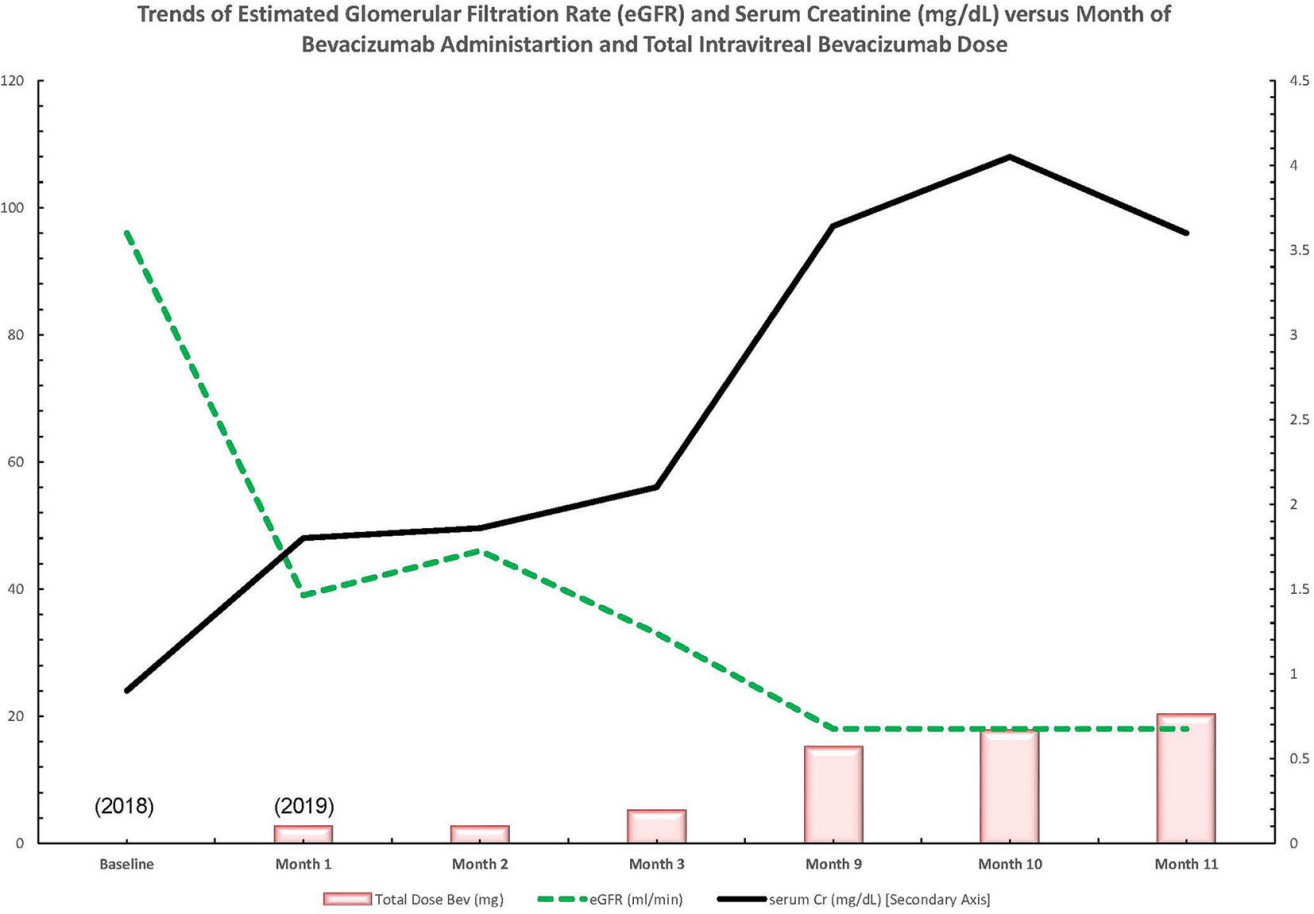
Trend of lab values over time in a patient with diabetic retinopathy treated with bevacizumab and subsequent thrombotic microangiopathy. Bev, bevacizumab; Cr, creatinine; dl, deciliter; eGFR, estimated glomerular filtration rate; mg, milligram; ml, milliliter; min, minute.

Early in 2019, the patient's serum creatinine rose to 1.44 mg/dl and then 1.86 mg/dl by 4/2019 (reference range: 0.7–1 mg/dl). In the latter half of 2019, the patient presented to nephrology with severely increased blood pressure, first in 9/2019 with a blood pressure of 214/107 mmHg and again in 10/2019 with a blood pressure of 236/108 mmHg; dyspnea; and severely worsened bilateral lower-extremity edema. At this time, the patient had an elevated serum creatinine of 3.6 mg/dl, as well as a microalbumin/creatinine ratio of >600 μg/mg (reference range: <30 μg/mg or mg/g) (none on baseline in 2018 and 359 μg/mg in 04/2019). A 24-h urine protein collection revealed that the patient had nephrotic range proteinuria with a total of 6.5 g of protein per day (reference range: <80 mg/24 h). Hypoalbuminemia had greatly worsened to 2.8 g/dl from a baseline of 3.8 g/dl in 4/2018 (reference range: 3.4–5.4 g/L). The patient's severe hypertension prompted admission for blood pressure control. After the patient's hypertension was controlled, a kidney biopsy was obtained given the rapid onset of renal dysfunction, worsening proteinuria, and accelerated hypertension (Figure 1).

**Figure 2:**
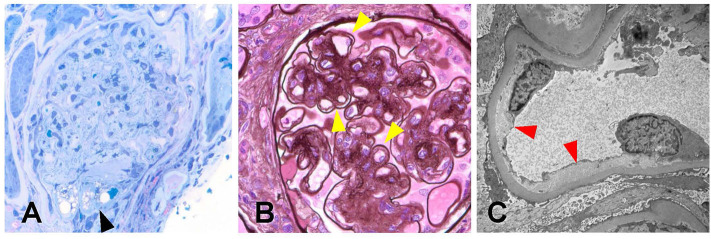
Biopsy findings in patient 1 with diabetic retinopathy and nephropathy treated with bevacizumab and subsequent thrombotic microangiopathy. **(A)** One glomerulus showed segmental luminal obliteration by insudates and lipid, with adherence to Bowman's capsule consistent with segmental glomerulosclerosis (arrowhead, methylene blue stain, 400×). **(B)** Few glomeruli demonstrated segmental duplication of glomerular basement membranes (arrowhead, Jones methenamine silver stain, 400×). **(C)** Ultrastructural analysis revealed segmental subendothelial electron lucent widening, with very early duplication of basement membrane material (arrowheads, 20,000×). The light and ultrastructural findings were consistent with chronic thrombotic microangiopathy.

From the biopsy samples, 33 glomeruli were identified, four of which were globally sclerotic. Three glomeruli contained lesions of segmental sclerosis characterized by luminal obliteration by insudates, foam cells, and lipid, with focal adherence to Bowman's capsule (Figure 2A). The glomeruli were normal in size with predominantly single-contoured capillary basement membranes with segmental double contours (Figure 2B) and patent capillary lumina. Mesangial areas showed diffuse and focal nodular expansion by matrix material with segmental mesangiolysis and microaneurysm formation. Few glomeruli displayed variable ischemic changes. No crescents or necrotizing features were present. There was moderate parenchymal scarring with mild interstitial inflammation. Arteries displayed moderate intimal fibrosis, and arterioles showed prominent afferent and efferent hyalinization. Immunofluorescence was negative for significant glomerular immune complex deposition.

Electron microscopy revealed glomerular basement membranes with normal trilaminar structure and global thickening (up to 1,440 nm). Segmentally, there was mild electron lucent, subendothelial widening with segmental glomerular basement membrane duplication and mesangial cell interposition. Focally within these areas, there was accumulation of flocculent and electron lucent debris with mild layering of new basement membrane material (Figure 2C). Mesangial areas were expanded by matrix material, and there was ~50% podocyte foot process effacement. The pathological findings showed a renal TMA in a background of diabetic nephropathy.

With the diagnosis of TMA, review of peripheral blood smears and laboratory parameters was undertaken. Peripherally, there were no schistocytes, and vitamin B12 level was 571 pg/ml (reference range: 300–950 pg/ml). ADAMTS13 was 117% of reference range activity (reference range: 50–160%). Severe ADAMTS13 deficiency was <5–10%. This ruled out any ADAMTS13 deficiency/thrombotic thrombocytopenic purpura. No diarrhea was noted, suggesting that there was no typical hemolytic uremic syndrome or evidence for the presence of Shiga toxin (reference range: undetectable). Platelets remained in normal range (reference range: 150,000–450,000/uL) despite hemoglobin level decline over the course of the year. Serum VEGF level on intravitreal anti-VEGF therapy was 34 pg/ml, which is near the lower limit of the reference range (reference range: 31–310 pg/ml). The presentation did not seem to fit the classical systemic presentation of an atypical hemolytic uremic syndrome but rather seemed to conform to a renal limited TMA as the biopsy suggested.

The patient's serum creatinine worsened to a level of 3.6–3.64 mg/dl (reference range: 0.7–1 mg/dl) a year after presentation. Intravitreal injections were discussed with the patient as a possible cause for TMA, but as of now, they are being continued due to the patient's severe visual impairment. The patient is now preparing for hemodialysis. Table 2 summarizes lab value trends for cases 1–3.

**Table 2:** A1c, glycated hemoglobin; ADAMTS13, a disintegrin and metalloproteinase thrombospondin motif #1, member # 13; B12 cyanocobalamin; dL, BL, baseline; deciliter; eGFR, estimated glomerular filtration rate; g, gram; L, liter; min, minute; mL, milliter; VEGF, vascular endothelial growth factor; VEGFi, VEGF inhibitor; uL, microliter.

**Lab value**	**Case 1 BL**	**Case 1 Post-VEGFi**	**Case 2 BL**	**Case 2 Post-VEGFi**	**Case 3 BL**	**Case 3 post VEGFi**
Age	56		43		77	
Gender	Male		Female		Female	
Identified ethnicity	Caucasian		Hispanic		Guyanese	
Total Dose of VEGFi	20 mg Bevacizumab (2018–2020)		7.5–15 mg bevacizumab		Ranibizumab (unknown quantity), 28 mg of aflibercept given	
Time frame of AKI in relation to initiation or changes to intravitreal VEGFi	2 years after starting bevacizumab 1 year after increasing frequency of injections		6 months after bevacizumab initiation		Year after changing from ranibizumab to aflibercept	
Serum Creatinine (mg/dL)	0.9 (2018)	1.8–3.6 (2019)	Normal	3.6 (2019)	1 (2019)	1.4 (2020)
eGFR (ml/min)	96 (2018)	54–18 (2019)	Not reported	25–30 (2019)	51 (2019)	37 (2020)
Hemoglobin (g/dL)	13 (2018)	7.7–11.1 (2019)	Not reported	Not reported	Normal (2019)	Normal (2020)
Platelets (/uL)	256,000(2018)	210–248,000 (2019)	Not reported	Not reported	Normal (2019)	Normal (2020)
Albumin (g/L)	3.8 (1/2019)	3.4 to 2.8 (11/2019)	Not reported	Not reported	Normal (2019)	Normal (2020)
Hemoglobin A1C (%)	10.4 (1/2019)	6.4–6.6 (11/2019)	Not reported	Not reported	5–5.7% (2019)	5–5.7% (2020)
Systolic blood pressure (mmHg)	134–177 (every 2–month VEGF inhibitor)	177–236 (every 1–month VEGF inhibitor eye injections)	150–160 (2019)	150–160 (2019)	Normal (2019)	150 (2020)
Diastolic blood pressure (mmHg)	74–86 (every 2–month VEGF inhibitor)	74–108 (every 1–month VEGF inhibitor eye injections)	90 (2019)	90 (2019)	Normal (2019)	100 (2020)
24 hour urine total protein (g/day)	Not reported	6.5 (10/2/2019)	Not reported	>3 (2019)	Not reported (2019)	0.8 (2020)
Urine microalbumin/Creatinine ratio (mcg/mg or mg/g)	360	>600 (9–11/2019)	Not reported	Not reported	<30 (2019)	800 (2020)
Serum VEGF level (pg/mL)	Not reported	34 (11/2019)	Not reported	Not reported	Not reported	Not reported
ADAMTS13 (%)	Not reported	117(11/2019)	Not reported	Not reported	Not reported	Not reported

### Case 2

A 43 year-old female with a history of type 2 diabetes mellitus had a subacute decline of her kidney function over 6 months, which was faster than expected for typical diabetic nephropathy. The treating physician noted that this occurred after the initiation of intravitreal bevacizumab. Her initial serum creatinine was reported only as normal, but her final serum creatinine was reported as 3.6 mg/dl (reference range: 0.7–1 mg/dl) with a eGFR <30 ml/min (stage IV CKD, G4, A3) (reference range: 90–120 ml/min). She had >3 g/day of proteinuria (reference range: <80 mg/day).

Given the standard bevacizumab dose of 1.25–2.5 mg every month, the estimated total dose she was exposed to is estimated to be up to 7.5–15 mg intravitreally over 6 months. She was noted to have accelerated worsening of her hypertension and nephrotic range proteinuria, but this was successfully controlled with blood pressure medications without improvement in her renal function. The worsening of blood pressure, proteinuria, and kidney function was noted to have occurred contemporaneously with initiating intravitreal bevacizumab for DR/DME. The patient had moderate hypertension at 150–160 mmHg systolic blood pressure but did not have clinically apparent malignant hypertension.

**Figure 3:**
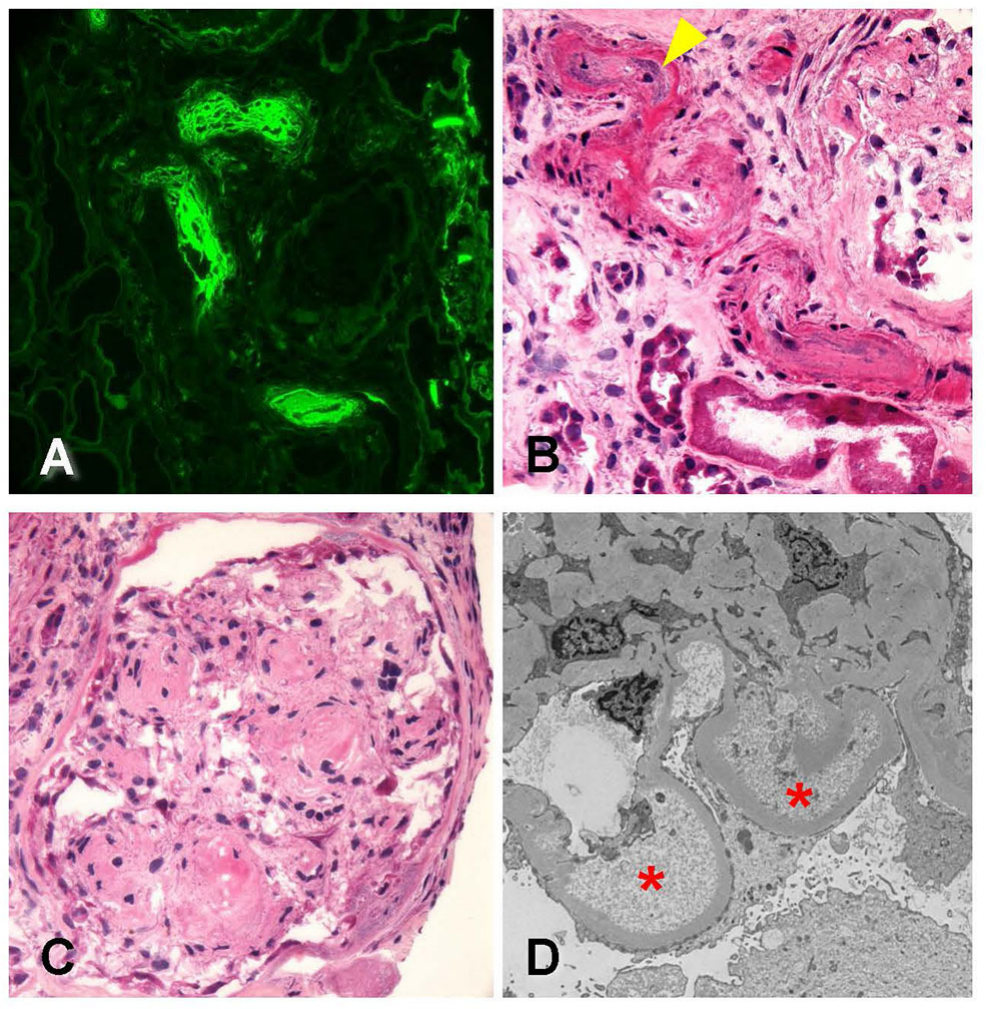
Biopsy findings in patient 2 with diabetic retinopathy and nephropathy treated with bevacizumab and subsequent thrombotic microangiopathy. **(A)** Immunofluorescence microscopy revealed scattered arterioles which displayed strong amorphous intraluminal and vessel wall staining for fibrinogen (400×). **(B)** Examination of hematoxylin–eosin (H&E)-stained sections from the frozen tissue demonstrated that the fibrin staining corresponded with changes of arteriopathy, including mucoid intimal thickening (arrowhead) and considerable luminal narrowing, consistent with acute thrombotic microangiopathy (400×). **(C)** Glomeruli showed changes of diffuse and nodular diabetic glomerulosclerosis (600×). **(D)** Ultrastructural analysis revealed glomerular basement membranes which showed prominent subendothelial electron lucent widening with accumulation of flocculent debris (20,000×). Overall, the findings were consistent with acute thrombotic microangiopathy.

The biopsy identified, overall, 29 glomeruli, eight of which were globally sclerotic. Glomeruli ranged in size from normal to enlarged with single-contoured capillary basement membranes and predominantly patent capillary lumina. One glomerulus displayed segmental luminal obliteration by insudates and lipid. Immunofluorescence staining revealed prominent staining for fibrinogen within these arterioles (Figure 3A). Arteries displayed mild to moderate intimal fibrosis, and arterioles had muscular hypertrophy, insudates, and mucoid intimal thickening with luminal narrowing and endothelial cell swelling (Figure 3B). Mesangial areas displayed diffuse and nodular expansion by matrix material (Figure 3C). No crescents or necrotizing features were noted. There was severe parenchymal scarring with mild interstitial inflammation.

Electron microscopy revealed glomerular basement membranes with normal trilaminar structure and global thickening (up to 1,210 nm). Segmentally, there were subendothelial lucencies with flocculent material as well as segmental mesangial cell interposition with double-contour formation (Figure 3D). Podocytes displayed subtotal foot process effacement. This suggested endothelial injury, a chronic TMA, and concomitant secondary focal and segmental sclerosis due to VEGF blockade. Table 2 summarizes lab value trends for cases 1–3.

### Case 3

A 77 year-old Guyanese female was referred to nephrology for worsening hypertension and proteinuria. She had had known type 1 diabetes mellitus for over 20 years with known DR and retinal vein disease. She also had a history of hypertension for the last 15 years well controlled on single-agent enalapril 10 mg once a day. She had prior urinalysis done yearly that showed trace protein. In the last few months, she was noticed to have increasing proteinuria of 800 mg over 24 h and worsening hypertension requiring enalapril to be increased to 20 mg twice daily and addition of amlodipine 10 mg daily. In addition, her kidney function had worsened from a serum creatinine of baseline 1.0 mg/dl (reference range: 0.7–1 mg/dl) (eGFR = 51 ml/min; reference range: 90–120 ml/min) to 1.4 mg/dl (eGFR = 37 ml/min). Her physical exam was consistent with a blood pressure of 150/100 mmHg and 1+ lower-extremity edema. Her medication list revealed no nephrotoxic agent and no herbal medications.

Her serological testing was negative for anti-nuclear antibody (ANA) (reference range: <1:20), lupus serologies (reference range: not detected), paraprotein workup (reference range: not detected), and anti-neutrophil cytoplasmic antibody (ANCA) and phospholipase A2 receptor antibody (reference range: not detected). Cell counts (white blood cells, hemoglobin, and platelets) were all within normal limits. Her complements were within normal range, lactate dehydrogenase was normal, and there was no decrease noted in haptoglobin. Her repeat urinary spot protein/creatinine ratio was 0.8. Her hemoglobin A1c had been in the 5.5–7% range (reference range: <5.7%) in the last few years. On further questioning, she mentioned she had been receiving ranibizumab for her DME for 4 years. In the last 1 year, she was switched to aflibercept 2 mg every 4 weeks for each eye, intravitreal for the first 3 months and then every 8 weeks following, leading to a total dose of 28 mg. As a result, a kidney biopsy was performed.

**Figure 4:**
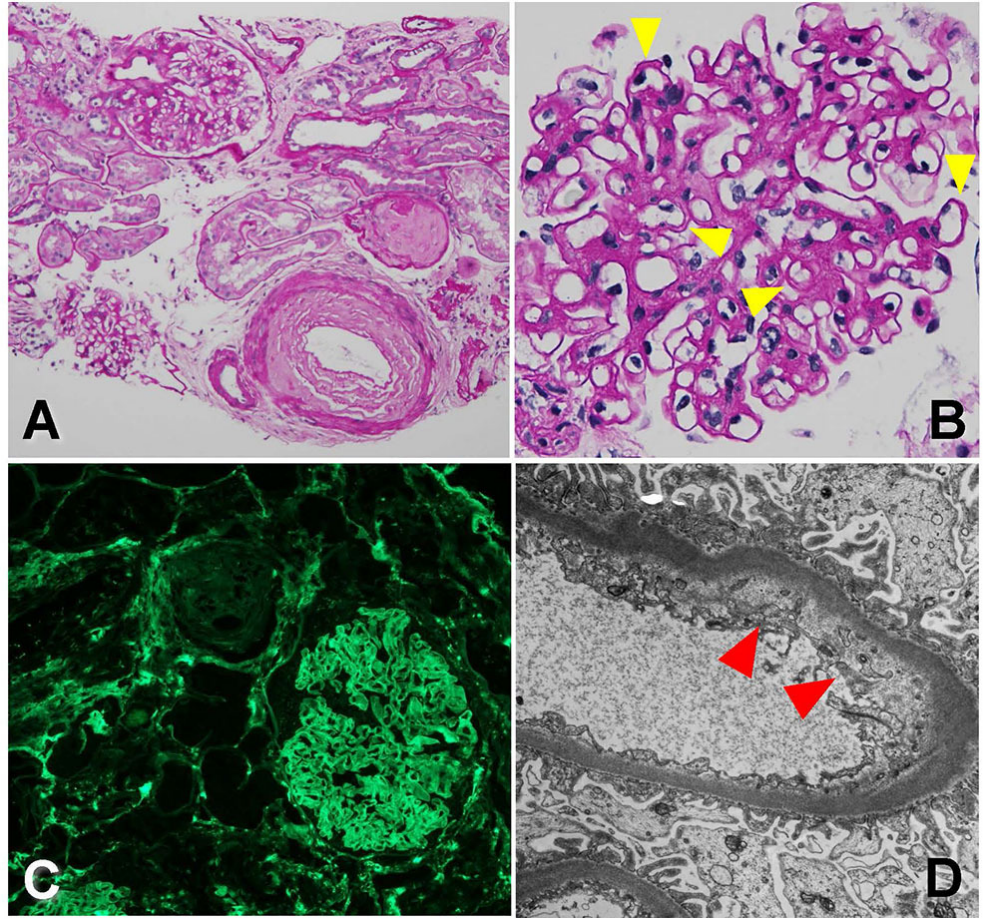
Biopsy findings showing arteriopathy and chronic thrombotic microangiopathy in patient 3. **(A)** There is severe arterial sclerosis, associated with focal global glomerulosclerosis, periodic acid-Schiff (PAS) stain, 200×. **(B)** Non-sclerosed glomeruli reveal irregular thickening and segmental remodeling of the capillary loops, with occasional double contours (yellow arrowheads), PAS stain, 400×. **(C)** There is dull reactivity for fibrin along the glomerular capillary walls on immunofluorescence microscopy, fluorescein isothiocyanate (FITC) stain, 200×. **(D)** On electron microscopy, glomerular capillary walls reveal subendothelial widening by electron lucent material (red arrowhead), 10,000×.

The biopsy was dominated by chronic changes, in the setting of severe arterial sclerosis [Figure 4A, periodic acid–Schiff (PAS) stain, 200×]. A large subcapsular scar containing 15 globally sclerosed glomeruli was found in one of the biopsy cores. Outside of this scar, there were up to 10 glomeruli, often revealing irregular thickening and segmental remodeling of the capillary loops, with occasional double-contour formation (Figure 4B, PAS stain, 400×, yellow arrowheads). The mesangium revealed mild expansion by matrix, without well-developed Kimmelstiel–Wilson nodules. Overall, there was about 40–50% tubular atrophy and interstitial fibrosis in this biopsy sample. No active glomerular or interstitial inflammation was noted. On immunofluorescence microscopy, no immune-type deposits were present, but there was dull reactivity for fibrin along the glomerular capillary walls [Figure 4C, fibrinogen fluorescein isothiocyanate (FITC) stain, 200×]. Glomerular capillary walls often revealed subendothelial widening by electron lucent material on electron microscopy (Figure 4D, 10,000×, red arrowheads).

Given signs of only early diabetic nephropathy on the kidney biopsy and with most of the changes noted to be related to endothelial and vascular damage, we attributed the findings to the anti-VEGF therapy this patient was exposed to over the last few years. The change in renal function and proteinuria timely fit with the initiation of aflibercept, and therefore, reverting back to the initial treatment (ranibizumab) for her DME was prudent. After discussion with the patient's treating retina specialists, she was taken off aflibercept and returned back to ranibizumab intravitreal treatment. Table 2 summarizes lab value trends for cases 1–3.

## FDA Adverse Report System Events

In addition to reviewing the published literature, we also reviewed the US FDA adverse event reporting system (FAERS) quarterly legacy data file (first quarter of 2010 to second quarter of 2019) for both aflibercept (Eylea®) and ranibizumab (Lucentis®) since the years they were approved specifically for intravitreal indications. Bevacizumab was not reviewed given the mixed results it would provide with use on oncology patients. The adverse event terms queried were proteinuria, renal failure acute, AKI, hypertension, thrombocytopenia nephritis, and TMA. Table 3 summarizes the data from the FAERS. Hypertension is the most common renal adverse event reported; other notable side effects include proteinuria. Few cases of TMA have been reported to the FDA from both agents. There are more cases reported of ranibizumab over aflibercept given the approval data of the latter being in 2016. Interestingly, most events happened in male patients for unknown reasons.

**Table 3:** Review of FDA FAERS for adverse events affecting Kidney by gender for Lucentis (ranibizumab) and Eylea (aflibercept).

**Name of Medication**	**Reaction**	**Male (*N* = 101) *n* (%)**	**Female (*N* = 160) *n* (%)**	**Missing (*N* = 144) *n* (%)**	**Overall (*N* = 405) *n* (%)**
Aflibercept (m = 97)	Hypertension	5 (4.95)	20 (12.50)	52 (36.11)	77 (19.01)
	Proteinuria	5 (4.95)	0 (0.00)	6 (4.17)	11 (2.72)
	Thrombocytopenia	0 (0.00)	0 (0.00)	6 (4.17)	6 (1.48)
	Renal Injury	0 (0.00)	0 (0.00)	2 (1.39)	2 (0.49)
	Thrombotic Microangiopathy	0 (0.00)	0 (0.00)	1 (0.69)	1 (0.25)
Ranibizumab(m = 308)	Hypertension	75 (74.26)	130 (81.25)	68 (47.22)	273 (67.41)
	Thrombocytopenia	7 (6.93)	8 (5.00)	3 (2.08)	18 (4.44)
	Thrombotic Microangiopathy	4 (3.96)	0 (0.00)	4 (2.78)	8 (1.98)
	Renal Injury	3 (2.97)	1 (0.63)	1 (0.69)	5 (1.23)
	Proteinuria	2 (1.98)	1 (0.63)	1 (0.69)	4 (0.99)

*-Adverse events reported as (Renal Failure, Renal Impairment, Renal Failure Acute, Renal Injury, Nephritis) presented as one group (RENAL INJURY)*.

-Percentage(%)= n/N^*^100.

## Discussion

There are 26 published cases showing worsening hypertension, proteinuria, and glomerular disease after intravitreal VEGF inhibition (2, 4, 32–46). Our group has published nine cases (2, 4, 45, 46). There are three more in this case series and one more under review. In total, there are 30 known cases demonstrating systemic toxicity after intravitreal VEGF inhibitor injections (2, 4, 32–46). See Table 1.

The cases presented in this manuscript show renal limited TMA in patients with poorly controlled diabetes and hypertension. The pattern of injury is exactly what is expected with VEGF blockade systemically. The timeline of initiation or increased dosing of intravitreal VEGF blockade fit the timeline of renal injury and proteinuria exacerbation, and this is what suggested the diagnosis clinically. The finding of pathognomonic lesions of VEGF blockade on kidney biopsy confirmed our clinical suspicion.

Other TMA presentations after intravitreal VEGF blockade in the literature are reviewed in Table 1 (4, 32, 39, 42, 43, 46) along with other published evidence (2, 4, 32–46). Other glomerular lesions such as collapsing glomerulopathy have been associated with intravitreal anti-VEGF agents as well (2, 4). cFSGS is a TMA-associated lesion that has been noted in conjunction with TMA presentations as was seen in case 2 in this series (48).

Tying the pathophysiology with mechanism, evidence of absorption, evidence of VEGF depletion, and clear biopsy findings has made these cases valuable. An important clinical lesson from these cases is that diabetic nephropathy *per se* cannot be invoked to account for an abrupt rise in serum creatinine. In addition, the secondary glomerular findings such as TMA and collapsing glomerulopathy are not features of diabetic nephropathy. It is likely that these renal pathological changes occur preferentially in proteinuric, hypertensive patients with preexisting renal disease. This is similar to preeclampsia, a naturally occurring disease model that approximates the pharmacologic phenomenon of VEGF blockade (49). This model of differential susceptibility to VEGF depletion has been suggested by a recently conducted South Korean study, showing that patients with more proteinuria at baseline were more likely to experience worsening proteinuria after intravitreal VEGF injections (47).

The FAERS database analysis suggests that hypertension might be the most common renal adverse event reported (Table 3). This is important as it might be the first sign of a systemic endothelial injury as seen with other anti-VEGF agents in the oncology literature.

These cases are extremely challenging to diagnose, and it is useful to consider the role of intravitreal VEGF blockade in every diabetic patient. The clinician needs to have a high index of suspicion to consider this diagnosis. The meticulous measurement of urine protein and albumin in addition to monitoring blood pressure changes is needed to document the effect of VEGF depletion on the kidney. Specialty consultation with a nephrologist in case of abrupt changes in renal parameters and monitoring patients receiving these agents closely are prudent recommendations.

Table 4 details clinical clues that raise the suspicion that intravitreal VEGF inhibition may be leading to renal or systemic toxicity. Recommendations for referral to specialty nephrology care are also listed in Table 4. They are a rise in serum blood urea nitrogen and creatinine by 25% or more acutely, an increase in blood pressure by 20 mmHg acutely, and an increase in urine protein-to-creatinine ratio by 25% or more after initiating intravitreal VEGF blockade (1).

**Table 4:** When to Consider Intravitreal VEGF Toxicity, Referral to Nephrology.

• Rapid worsening of renal function (25% rise in BUN, Cr over short time)
• Unexplained changes in blood pressure (>20 mmHg over short time)
• Rapid or unexpected change in proteinuria (>25% rise over short time)
• If any of above occur: Check urinalysis, Urine protein to Creatinine ratio, Refer to nephrology
• If suspicion of intravitreal anti VEGF renal toxicity: consider decreasing dose, change to lower potency VEGF inhibitors (like ranibizumab)

*mcg, micrograms; TMA, thrombotic microangiopathy*.

There are comprehensive reviews that highlight the lesions and clinical manifestations seen after intravitreal VEGF blockade (1, 2). The utility of this report is to document three examples of the prototypical renal lesions resulting from systemic VEGF blockade in patients receiving VEGF inhibitors intravitreally. Currently, the only known risk factors for worsening hypertension and proteinuria after intravitreal VEGF injection are preexisting hypertension and proteinuria at baseline.

There is no specific guidance regarding the treatment of intravitreal VEGF inhibitor-associated glomerular lesions at this time. In our experience, oral corticosteroids for treatment of cFSGS lesions induced while patients were getting intravitreal VEGF blockade were not uniformly successful. Given the emerging evidence of efficacy of complement blockade in some secondary forms of TMA/atypical hemolytic uremic syndrome, use of complement factor 5 blockade may be a therapeutic option (50).

Ultimately, DR and AMD can lead to irreversible visual deterioration and blindness (1). Intravitreal VEGF blockade in ameliorating these diseases has been important. It is important to note that the rate of renal events occurring with intravitreal VEGF blockade requires further study. We acknowledge the importance of intravitreal VEGF blockade but propose that patients receiving intravitreal VEGF blockade require close monitoring (1). If there are concerns regarding renal sequelae after intravitreal VEGF blockade, prompt referral to nephrological care is crucial (1). The importance of the ophthalmologist and retina specialists, who are closely monitoring patients with retinal pathology, cannot be overstated. A registry to track these events can suggest how common the events are, and controlled observational trials following pharmacokinetic data would be helpful. A new era of ophthalmological and nephrological collaboration in research and patient care is clearly needed to fully investigate the systemic risks of intravitreal VEGF blockade.

## Data Availability Statement

The original contributions presented in the study are included in the article, further inquiries can be directed to the corresponding author/s.

## Ethics Statement

Documented (written) informed consent was obtained from the individuals in cases 1–3, for the publication of any potentially identifiable images or data included in this article.

## Author Contributions

RH: lead writing of case series. N-TT: contributed to case 1. SP: contributed to case 2. JH: pathology to case 2. KJ: contributed to case 3. RP: ERAS search. US: contributed to introduction. LG, OW, MB, KK-Z, AP, and AA: editing of manuscript. VB: pathology of case 3. BK: editing of manuscript from ophthalmology perspective. IK: senior author. All authors contributed to the article and approved the submitted version.

## Conflict of Interest

AA has served as principal investigaor or co-invesitgator for NIH/NIAID, NeuroRx Pharma, Pulmotect, Blade Therapeutics, Novartis, Takeda, Humanigen, Eli-Lliy, PTC Therpeutics, OctaPharma, Fulcrum Therapeutics, and Alexion; and has been a consultant or speaker for BMS, Pfizer, BI, Portola, Sunovion, Mylan, Alexion, Astra Zeneca, Novartis, Nabriva, Paratek, Bayer, Tetraphase, Achogen, and LaJolla. The remaining authors declare that the research was conducted in the absence of any commercial or financial relationships that could be construed as a potential conflict of interest.
